# Associations of *Helicobacter pylori* with metabolic dysfunction-associated steatotic liver disease and related conditions: cross-sectional results from the Hispanic Community Health Study/Study of Latinos

**DOI:** 10.1016/j.lana.2024.100953

**Published:** 2024-11-30

**Authors:** Christian S. Alvarez, Robert C. Kaplan, M. Constanza Camargo, M. Larissa Avilés-Santa, Martha Daviglus, Olga Garcia-Bedoya, Carmen R. Isasi, Maria S. Pattany, Bharat Thyagarajan, Gregory A. Talavera, Barry I. Graubard, Katherine A. McGlynn

**Affiliations:** a Division of Intramural Research, National Institute on Minority Health and Health Disparities, NIH, Rockville, MD, USA; b Department of Epidemiology and Population Health, Albert Einstein College of Medicine, Bronx, NY, USA; c Public Health Sciences Division, Fred Hutchinson Cancer Research Center, Seattle, WA, USA; d Division of Cancer Epidemiology and Genetics, National Cancer Institute, Rockville, MD, USA; e Division of Clinical and Health Services Research, National Institute on Minority Health and Health Disparities, Rockville, MD, USA; f Institute for Minority Health Research, University of Illinois Chicago, Chicago, IL, USA; g Academic Internal Medicine and Geriatrics, Department of Medicine, University of Illinois Chicago, Chicago, IL, USA; h Department of Psychology, College of Arts and Sciences, University of Miami, Coral Gables, Florida; i Department of Laboratory Medicine and Pathology, University of Minnesota, Minneapolis, MN, USA; j South Bay Latino Research Center, Department of Psychology, San Diego State University, San Diego, CA, USA

**Keywords:** MASLD, *Helicobacter pylori*, Hispanic/Latino

## Abstract

**Background:**

Hispanic/Latino populations have been reported to have high rates of both metabolic dysfunction-associated steatotic liver disease (MASLD) and *Helicobacter pylori* infection. Several observational studies, predominantly from Asian populations, have suggested a link between these conditions. Thus, the primary objective of the current study was to examine the association between *H. pylori* and MASLD and secondarily, to assess its association with related conditions in the Hispanic Community Health Study/Study of Latinos.

**Methods:**

In this cross-sectional study, a total of 16,144 participants with baseline data on *H. pylori* serology were included. Based on weighted statistics, the median age was 40 years [interquartile range (IQR): 28, 52]; 52.2% women (n = 9661) and 47.8% men (n = 6483). Participants’ Hispanic/Latino heritage included 37.6% Mexicans (n = 6397), 20.1% Cubans (n = 2307), 15.8% Puerto Ricans (n = 2663), 10.0% Dominicans (n = 1447), 7.4% Central Americans (n = 1710), 4.9% South Americans (1052). MASLD was estimated using the Fatty Liver Index (FLI) and the Hepatic Steatosis Index (HSI). Other conditions examined were obesity, central obesity, diabetes and metabolic syndrome. Multivariable logistic regression models were used to calculate the ratios of (adjusted) prevalences (RP) and 95% confidence intervals (CI) for the overall association of *H. pylori* seropositivity with MASLD and related conditions. Analyses were also stratified by Hispanic/Latino heritage.

**Findings:**

The overall prevalence of MASLD ranged from 47% (FLI) to 65% (HSI). After accounting for age, sex, education, and other key variables, the analysis found a modest association between *H. pylori* seropositivity and MASLD as determined by HSI (RP: 1.06, 95% CI: 1.02–1.10) overall, and among individuals of Puerto Rican and Mexican heritages. Furthermore, an overall association between *H. pylori* seropositivity and obesity was observed (RP: 1.09, 95% CI: 1.02–1.16).

**Interpretation:**

This study provides support for a positive association of *H. pylori* seropositivity with MASLD and obesity among Hispanic/Latino populations. However, given the exploratory nature of these findings, caution is warranted in their interpretation. Further research is necessary to establish causality and examine potential mechanisms of these associations.

**Funding:**

The Hispanic Community Health Study/Study of Latinos was carried out as a collaborative study supported by contracts from the 10.13039/100000050National Heart, Lung, and Blood Institute (NHLBI) to the 10.13039/100006808University of North Carolina (N01-HC65233), 10.13039/100006686University of Miami (N01-HC65234), 10.13039/100007319Albert Einstein College of Medicine (N01-HC65235), 10.13039/100007059Northwestern University (N01-HC65236), 10.13039/100007099San Diego State University (N01-HC65237), and 10.13039/100008522University of Illinois at Chicago (HHSN268201300003I). The following Institutes/Centers/Offices contribute to the HCHS/SOL through a transfer of funds to the NHLBI: 10.13039/100006545National Institute on Minority Health and Health Disparities, United States, the National Institute of Deafness and Other Communications Disorders, the 10.13039/100000072National Institute of Dental and Craniofacial Research, the 10.13039/100000062National Institute of Diabetes and Digestive and Kidney Diseases, the 10.13039/100000065National Institute of Neurological Disorders and Stroke, and the 10.13039/100000063Office of Dietary Supplements. This study was also funded in part by the 10.13039/100030692Intramural Research Program of the 10.13039/100000054National Cancer Institute.

**Research in context**

**Evidence before this study**

We systematically searched PubMed for studies examining associations between *Helicobacter pylori* with metabolic-dysfunction associated steatotic liver disease (MASLD). Our main search terms included combinations of “*Helicobacter pylori*”, “nonalcoholic fatty liver disease”, “metabolic dysfunction-associated steatotic liver disease”, “metabolic syndrome”, “obesity”, and “insulin resistance”. The search covered the period from January 1, 2015 to August 31, 2023, and was restricted to English-language studies.

Recent evidence suggests an association between *H. pylori* with MASLD as well as other metabolic conditions. A recent meta-analysis, predominantly including cross-sectional studies in Asian populations, reported an association between *H. pylori* infection and MASLD, with a pooled odds ratio of 1.28 (95% confidence interval: 1.18, 1.38). However, findings have been mixed, as some studies have not observed this association, leading to inconclusive results.

**Added value of this study**

This study advances understanding of the association between *H. pylori* infection and MASLD by examining these relationships within a large, diverse sample of Hispanic/Latino individuals in the United States – an understudied population with high rates of both conditions. Prior research has largely focused on Asian populations and has produced mixed results, leaving gaps in understanding how these associations may vary across populations. Our findings provide valuable insights into the potential link between *H. pylori* and MASLD in this specific population, underscoring the importance of investigating infectious contributors to metabolic health.

**Implications of all the available evidence**

Eradication of *H. pylori* might represent a potential strategy for the prevention of MASLD. However, further large-scale longitudinal studies are warranted to confirm these findings and to elucidate the underlying pathogenic mechanisms.

## Introduction

Metabolic dysfunction-associated steatotic liver disease (MASLD), formerly called nonalcoholic fatty liver disease (NAFLD) is the most common form of chronic liver disease in many regions of the world, and is associated with hepatocellular carcinoma.^1^ MASLD is also closely related to other conditions, such as metabolic syndrome (MetSyn), obesity, central adiposity, and diabetes, all of which have increased in prevalence over time.^1^ In the US, the overall prevalence of MASLD (as defined by controlled attenuation parameter derived via transient elastography) is ∼48% in the general population, but disproportionally affects (∼56%) Hispanic/Latino individuals.^2^ A previous study conducted in the Hispanic Community Health Study/Study of Latinos (HCHS/SOL) found that the prevalence of suspected MASLD, as defined by elevated transaminase levels, varied by heritage, ranging from 15% in Dominicans to 22% in Mexicans.^3^

*Helicobacter pylori* is the most common gastrointestinal infection worldwide.^4^ Recent HCHS/SOL study results shows a 57% overall seroprevalence of *H. pylori* infection, which varied by heritage (47% in Puerto Ricans to 72% in Central Americans).^5^ A recent meta-analysis, predominantly including cross-sectional studies in Asian populations, have reported an *H. pylori* infection-MASLD association with a pooled odds ratio (OR) = 1.28 (95% CI: 1.18, 1.38).^6^ The Third National Health and Nutrition Examination Survey (NHANES) reported an increased risk of MASLD in *H. pylori* seropositive patients that were cytotoxin-associated gene A (cagA)-negative.^7^ While a study in Guatemala found no overall associations of *H. pylori* and other species of *Helicobacter* with MASLD, there was a suggestion that seropositivity for *H. pylori* cagA and vacuolating cytotoxin A (vacA) were associated with MASLD. Furthermore, antibodies to other *H. pylori* antigens (hyuA and ureA) were associated with obesity and diabetes, respectively.^8^ Based on these inconsistent results, an association between steatotic liver disease and *H. pylori* infection remains uncertain.

To our knowledge, only one small hospital-based study (n = 270) among U.S. Hispanic individuals previously examined the association of *H. pylori* infection and MASLD.^9^ Evaluating this association in ethnically diverse Hispanic/Latino populations with a high prevalence of *H. pylori* and a growing prevalence of MASLD provides key data to better understand whether these conditions are linked. Therefore, the primary objective of this study was to assess the association between *H. pylori* and MASLD within the HCHS/SOL study, and secondarily, to assess its association with related conditions.

## Methods

### Study population

Detailed information on the HCHS/SOL has been published.^10^ Briefly, HCHS/SOL is a cohort study designed to evaluate risk factors for chronic disease in diverse Hispanic/Latino populations. The HCHS/SOL study recruited 16,415 individuals aged 18–74 years from randomly selected households in four U.S. urban communities (San Diego, CA; Chicago, IL; Miami, FL; Bronx, NY) during 2008–2011. Questionnaires at the baseline examination were used to obtain information on a variety of factors, including but not limited to demographics, health/medical history, social/language acculturation, and smoking habits. Clinical assessments such as dental exams were also performed at baseline, and a fasting sample of blood was collected. The HCHS/SOL was approved by institutional review boards at each participating institution and written informed consent was obtained from all participants.

We conducted a cross-sectional study using baseline data collected at the HCHS/SOL initial visit on 16,144 individuals with available *H. pylori* serology. This analysis was approved by the HCHS/SOL Committee of Ancillary studies (AS#2016.08) and was exempted from institutional review board evaluation by the National Institutes of Health Office of Human Subjects Research Protection.

### Steatotic liver disease and fibrosis definitions

MASLD is a composite condition that was defined by liver steatosis scores, using validated indices, including the fatty liver index (FLI) and the hepatic steatosis index (HSI). FLI was calculated as

FLI = (e ^0.953^^loge (triglycerides) + 0.139^^BMI + 0.718^^loge (ggt) + 0.053^^waist circumference − 15.745^)/(1 + e ^0.953^^loge (triglycerides) + 0.139^^BMI + 0.718^^loge (ggt) + 0.053^^waist circumference − 15.745^) ∗ 100.^11^

An FLI ≥60 was considered elevated. HSI was computed as HSI = 8 × (ALT/AST ratio) + BMI (+2, if female; +2, if diabetes mellitus).^12^ An HSI score >36 was considered elevated. In contrast to the traditional definition of NAFLD, MASLD does not require the exclusion of individuals with excessive alcohol consumption or chronic HBV or HCV infections.^13^

In addition, suspected MASLD was defined as having an elevated AST or ALT level (men: AST >37 IU/mL or ALT >40 IU/mL; women: AST or ALT >31 IU/mL).^3^

Liver fibrosis indices were calculated as markers of disease severity, including fibrosis-4 (FIB-4) and AST-to-Platelet Ratio Index (APRI). FIB-4 score was calculated as: (age [years] ∗AST)/(platelet count [10^9^/L] ∗ ALT^(0.5)^). A FIB-4 score >2.67 was considered elevated.^14^ APRI score was calculated as: ([AST/upper limit of normal]/platelet count [10^9^/L]) × 100. An APRI score >1.5 was considered elevated.^14^

### Metabolic conditions definitions

Metabolic conditions of interest included obesity (body mass index [BMI] ≥30 kg/m^2^), abdominal obesity (waist circumference men: ≥94 cm, women: ≥80 cm, based on cut-offs suggested by the International Diabetes Federation)^15^ diabetes (defined as fasting plasma glucose ≥126 mg/dL, 2-h post-load plasma glucose ≥200 mg/dL, HbA1C ≥6.5%, or documented use of hypoglycaemic agents based on the American Diabetes Association definitions)^16^ and metabolic syndrome (defined by three or more of the following criteria: 1) waist circumference ≥102 cm in men and ≥88 cm in women; 2) triglyceride level ≥150 mg/dL; 3) reduced HDL-C levels <40 mg/dL in men and <50 mg/dL in women; 4) blood pressure ≥130 mmHg systolic and/or ≥85 mmHg diastolic, and/or the individual was receiving lowering blood pressure medication; and 5) fasting glucose level ≥100 mg/dL and/or the individual was receiving medication for lowering the blood glucose, based on the Adult Treat Panel III (ATP III) National Cholesterol Education Program cut-offs).^17^

### Laboratory assessment for *H. pylori*

As previously reported,^5^ anti-*H. pylori* immunoglobulin G antibodies were measured in baseline plasma using a commercial whole-cell enzyme-linked immunosorbent assay kit (GastroPanel® *Helicobacter pylori,* Biohit, Helsinki, Finland) following the manufacturer's instructions. Seropositivity was defined as titers >30 EIU.

### Statistical analysis

The seroprevalence of *H. pylori* and prevalences of MASLD and metabolic conditions were calculated for all participants together, and by Hispanic/Latino heritage. Weighted descriptive statistics were computed for all individuals, and by MASLD and metabolic conditions status. We conducted a complete case analysis, excluding participants with missing values for any of the variables included in our models. Predictive margins from multivariable logistic regression models were used to estimate adjusted prevalences of MASLD and metabolic conditions by *H. pylori* seropositivity. Associations were expressed as ratios of adjusted prevalences (RP) and 95% confidence intervals (CI). Models were adjusted for key confounders, including age, self-report sex assigned, or recorded at birth (hereafter referred to as sex), education, nativity, SASH (short acculturation scale for Hispanics) language acculturation, cigarette smoking, number of missing teeth, number of doctor's visits, ferritin levels and BMI (except for obesity, central obesity and FLI). The majority of these confounders were identified as *H. pylori* determinants in our previous study.^5^ The covariables with the highest proportion of missing data were missing teeth (7.4%), doctor visits (2.0%), ferritin (1.8%), nativity (0.7%) and language acculturation (0.6%). The proportions of missing data for the liver and metabolic conditions were less than 1.5%. Interaction terms were added to the final models and significance was evaluated using the Wald test. Interaction tests were pre-specified to explore potential differences in the association between *H. pylori* seropositivity and MASLD across diverse populations. Analyses were also stratified by Hispanic/Latino heritage, including those who self-identified as having a Mexican, Puerto Rican, Cuban, Dominican, South American or Central American background. No adjustment for multiple comparisons was made as most of the associations examined were secondary objectives or exploratory analyses. All p values are two-sided and a p value ≤ 0.05 was considered statistically significant. All analyses were performed using sample weights and the other aspects of the complex sample design via the survey analysis procedures in SAS 9.4 software (SAS Institute Inc., Cary, NC) and SUDAAN 11.0.3 software (Research Triangle Institute, Research Triangle Park, NC). Statistical analyses were conducted in 2023 according to a predefined statistical analysis plan in the study proposal, which was approved by the HCHS/SOL Publication Committee on January 21, 2021. The study proposal is available on the HCHS/SOL website upon registration at: https://sites.cscc.unc.edu/hchs/node/10388.

### Role of funding source

The funding source had no role in the design, conduct, or reporting of the study. Its content are solely the responsibility of the author and do not necessarily represent the official views of the NIH.

## Results

Table 1 shows selected baseline characteristics of the population by liver disease, and Table 2 displays these characteristics by metabolic condition status. Overall, among 16,144 individuals (recruited between 2008 and 2011), 52% were women (n = 9661), nearly 32% reported having less than high school education (n = 6103) and 77% were foreign born (n = 13,235). In addition, approximately 32% reported not seeing a doctor in the last year (n = 4446), and 21% reported currently smoking (n = 3099). Median age was similar among persons with most liver diseases and metabolic conditions (ranging from 39 [interquartile range (IQR): 29, 50] to 43 [IQR: 32, 55]) (Tables 1 and 2), except for persons with diabetes or elevated FIB-4 score, which ages were higher than persons with the other conditions (55 years [IQR: 45, 64] and 60 years [IQR: 49, 67], respectively; Tables 1 and 2). For most liver diseases, the proportion of men affected was greater than women affected. For metabolic conditions, the opposite was true, with the proportion of women affected being greater than the proportion of men. The proportion of individuals with less than a high school education was higher among those with diabetes compared to the rest of the conditions. Individuals with an elevated FIB-4 score had a high proportion of cigarette use as well as a higher proportion of >3 health-care provider (HCP) visits in the last year. Similarly to individuals with elevated FIB-4 score, participants with diabetes reported a greater proportion of HCP visits in the last year (51.5%).

**Table 1:** Selected characteristics of the analytical sample overall and by liver disease.

		Liver disease				
	Overall N = 16,144	Elevated FLI N = 7964	Elevated HSI N = 11,050	Suspected MASLD N = 3228	Elevated FIB-4 score N = 293	Elevated APRI score N = 697
	No. (%^a^)	No. (%^a^)	No. (%^a^)	No. (%^a^)	No. (%^a^)	No. (%^a^)
***H. pylori* seropositivity**	9877 (57.0)	4944 (58.9)	6907 (59.2)	1999 (58.3)	170 (54.3)	417 (55.4)
**Age, median (Q1, Q3)**	40 (28, 52)	43 (33, 54)	42 (31, 53)	39 (29, 50)	60 (49, 67)	43 (32, 54)
**Sex**						
Female	9661 (52.2)	4462 (47.0)	6846 (53.5)	1689 (41.2)	121 (30.7)	348 (39.4)
Male	6483 (47.8)	3502 (53.0)	4204 (46.5)	1539 (58.8)	172 (69.3)	349 (60.6)
Missing	0	0	0	0	0	0
**Heritage**						
Central American	1710 (7.4)	842 (7.0)	1210 (7.6)	378 (8.4)	25 (4.3)	73 (6.2)
South American	1052 (4.9)	437 (3.9)	659 (4.3)	204 (4.5)	6 (1.4)	33 (3.2)
Mexican	6397 (37.6)	3292 (39.0)	4508 (38.6)	1437 (43.5)	89 (21.0)	273 (37.2)
Cuban	2307 (20.1)	1118 (20.3)	1512 (19.6)	405 (18.2)	46 (32.7)	84 (18.3)
Puerto Rican	2663 (15.8)	1432 (17.5)	1855 (16.4)	491 (14.1)	107 (32.1)	174 (22.6)
Dominican	1447 (10.0)	569 (8.3)	933 (9.7)	203 (7.3)	12 (6.6)	34 (6.6)
Others	490 (4.1)	236 (4.0)	326 (3.8)	93 (4.0)	6 (2.0)	22 (6.0)
Missing	78	38	47	17	2	4
**Education level**						
Less than high school	6103 (32.4)	3239 (35.7)	4314 (33.9)	1220 (31.5)	149 (44.6)	301 (38.5)
High school or equivalent	4123 (28.3)	1956 (27.0)	2793 (27.9)	872 (30.1)	67 (25.4)	198 (32.4)
Greater than high school or equivalent	5836 (39.4)	2730 (37.4)	3889 (38.2)	1118 (38.4)	75 (30.1)	194 (29.2)
Missing	82	39	54	18	2	4
**Nativity**						
US born (50 states + DC)	2799 (22.8)	1367 (22.0)	1826 (21.3)	561 (22.2)	45 (14.1)	143 (26.4)
Foreign born (<10 years in US)	3749 (27.8)	1560 (23.0)	2345 (25.6)	722 (28.8)	40 (17.4)	118 (20.2)
Foreign born (≥10 years in US)	9486 (49.5)	4989 (55.0)	6816 (53.1)	1925 (49.0)	207 (68.4)	433 (53.3)
Missing	110	48	63	20	1	3
**Acculturation SASH_LANG score**^b^						
Low (1–2)	10,592 (59.5)	5261 (60.4)	7424 (61.7)	2126 (60.3)	186 (63.1)	437 (58.0)
High (≥3)	5462 (40.5)	2660 (39.6)	3568 (38.3)	1086 (39.7)	104 (36.9)	254 (42.0)
Missing	90	43	58	16	3	6
**BMI**						
<18.5	126 (1.2)	0 (0.0)	1 (0.0)	12 (0.5)	3 (1.3)	3 (0.9)
18.5–<25	3132 (22.0)	103 (1.3)	269 (2.5)	351 (11.2)	68 (26.2)	105 (14.8)
25–<30	6022 (37.2)	1889 (23.8)	4005 (36.8)	1050 (33.4)	96 (33.0)	209 (29.9)
≥30	6805 (39.7)	5972 (74.9)	6775 (60.7)	1803 (54.8)	123 (39.5)	378 (54.3)
Missing	59	0	0	12	3	2
**Smoking status**						
Never	9781 (61.4)	4501 (56.5)	6688 (60.7)	1904 (58.7)	130 (45.2)	366 (53.2)
Former	3184 (17.3)	1910 (21.7)	2406 (19.3)	684 (19.1)	71 (21.7)	151 (17.4)
Current	3099 (21.3)	1509 (21.8)	1905 (20.0)	620 (22.3)	89 (33.0)	172 (29.3)
Missing	80	44	51	20	3	8
**Ferritin (μg/L)**						
<12	15,300 (96.7)	7685 (97.5)	10,495 (96.7)	3140 (98.2)	290 (98.5)	685 (98.6)
≥12	559 (3.3)	219 (2.5)	373 (3.3)	55 (1.8)	3 (1.5)	8 (1.4)
Missing	285	60	182	33	0	4
**# of Doctor/HCP visits in the last year**						
0	4446 (31.7)	2087 (30.3)	2917 (30.4)	960 (33.6)	49 (17.7)	163 (28.9)
1	2535 (16.2)	1133 (14.8)	1678 (15.8)	487 (16.2)	30 (10.9)	94 (13.7)
2–3 times	3889 (24.1)	1850 (23.5)	2626 (23.9)	731 (22.6)	62 (22.8)	162 (23.6)
>3	4947 (28.0)	2741 (31.3)	3616 (29.9)	989 (27.6)	147 (48.6)	261 (33.8)
Missing	327	153	213	61	5	17
**Total number of missing teeth**						
0	4617 (40.9)	1873 (34.2)	2828 (37.0)	893 (39.3)	19 (9.3)	130 (33.1)
1–4	5450 (34.4)	2778 (36.4)	3850 (35.3)	1157 (35.9)	77 (25.9)	234 (34.1)
5–8	2276 (11.6)	1236 (13.5)	1688 (13.0)	469 (12.3)	52 (21.9)	114 (13.6)
≥9	2602 (13.1)	1437 (15.9)	1898 (14.8)	489 (12.6)	89 (42.9)	132 (19.2)
Missing	1199	640	786	220	56	87

Abbreviations: FLI, Fatty liver index; HSI, Hepatic Steatosis Index; MASLD, Metabolic dysfunction-associated fatty liver disease; FIB-4, Fibrosis-4; APRI, AST to Platelet ratio index.

Missing data for liver conditions: FLI (1.3%); HSI (0.4%); Suspected MASLD (0.0%); Elevated FIB-4 (0.8%); Elevated APRI (0.9%).

a Percentages are weighted by the sample weights.

b Short acculturation scale for Hispanics, language subscale.

**Table 2:** Selected characteristics of the analytical sample by metabolic condition.

	Metabolic condition			
	Obesity N = 6805	Abdominal obesity N = 9789	Diabetes N = 3176	Metabolic syndrome N = 6119
	No. (%^a^)	No. (%^a^)	No. (%^a^)	No. (%^a^)
***H. pylori* seropositivity**	4153 (58.3)	5990 (57.8)	2038 (61.3)	3894 (60.8)
**Age, median (Q1, Q3)**	42 (31, 53)	43 (32, 55)	55 (45, 64)	49 (38, 59)
**Sex**				
Female	4400 (56.0)	7380 (69.0)	1964 (55.0)	3897 (54.5)
Male	2405 (44.0)	2409 (31.0)	1212 (45.0)	2222 (45.5)
Missing	0	0	0	0
**Heritage**				
Central American	710 (7.1)	985 (6.9)	298 (6.9)	645 (7.3)
South American	361 (3.7)	526 (3.9)	131 (3.1)	311 (4.0)
Mexican	2725 (36.9)	4058 (38.5)	1286 (36.5)	2364 (35.1)
Cuban	874 (19.0)	1345 (19.7)	401 (21.6)	963 (23.5)
Puerto Rican	1288 (18.6)	1712 (17.2)	692 (19.5)	1165 (18.3)
Dominican	593 (10.2)	837 (9.9)	262 (9.6)	473 (8.0)
Others	226 (4.4)	289 (3.9)	59 (2.8)	158 (3.8)
Missing	28	37	47	40
**Education level**				
Less than high school	2689 (34.3)	3864 (34.5)	1547 (45.5)	2709 (38.8)
High school or equivalent	1719 (28.0)	2388 (27.0)	660 (22.8)	1388 (25.1)
Greater than high school or equivalent	2367 (37.7)	3495 (38.5)	920 (31.7)	1979 (36.1)
Missing	30	42	49	43
**Nativity**				
US born (50 states + DC)	1315 (25.0)	1677 (22.5)	335 (11.9)	834 (17.4)
Foreign born (<10 years in US)	1294 (22.0)	2025 (23.8)	477 (18.7)	1181 (23.4)
Foreign born (≥10 years in US)	4155 (53.0)	6034 (53.7)	2312 (69.4)	4053 (59.1)
Missing	41	53	52	51
**Acculturation SASH_LANG score**^b^				
Low (1–2)	4345 (57.4)	6583 (61.3)	2303 (73.0)	4336 (67.2)
High (≥3)	2423 (42.6)	3162 (38.7)	824 (27.0)	1741 (32.8)
Missing	37	44	49	42
**BMI**				
<18.5	0 (0.0)	0 (0.0)	3 (0.1)	2 (0.0)
18.5–<25	0 (0.0)	439 (4.2)	297 (9.6)	298 (4.4)
25–<30	0 (0.0)	3006 (29.7)	1008 (33.4)	1892 (31.0)
≥30	6805 (100.0)	6315 (66.1)	1842 (56.8)	3898 (64.6)
Missing	0	29	26	29
**Smoking status**				
Never	4041 (59.6)	6066 (62.5)	1774 (56.4)	3520 (56.6)
Former	1559 (20.1)	2030 (18.3)	851 (25.5)	1438 (21.9)
Current	1171 (20.3)	1649 (19.1)	501 (18.0)	1118 (21.5)
Missing	34	44	50	43
**Ferritin (μg/L)**				
<12	6458 (96.5)	9196 (95.6)	3067 (97.6)	5886 (97.8)
≥12	241 (3.5)	416 (4.4)	83 (2.4)	159 (2.2)
Missing	106	177	26	74
**# of Doctor/HCP visits in the last year**				
0	1691 (29.4)	2293 (26.8)	485 (17.9)	1401 (27.2)
1	984 (15.2)	1428 (15.2)	276 (8.1)	768 (12.7)
2–3 times	1605 (23.6)	2406 (24.5)	732 (22.5)	1421 (23.0)
>3	2386 (31.7)	3468 (33.5)	1575 (51.5)	2393 (37.0)
Missing	139	194	108	136
**Total number of missing teeth**				
0	1736 (37.3)	2351 (35.2)	425 (18.2)	1071 (26.4)
1–4	2334 (34.5)	3306 (34.5)	963 (32.9)	1985 (34.9)
5–8	1041 (12.8)	1527 (13.8)	576 (17.8)	1049 (16.2)
≥9	1188 (15.3)	1808 (16.5)	815 (31.1)	1388 (22.5)
Missing	506	797	397	626

Missing data for metabolic conditions: Obesity (0.4%); Abdominal Obesity (0.3%); Diabetes (0.0%); Metabolic Syndrome (0.0%).

a Percentages are weighted by the sample weights.

b Short acculturation scale for Hispanics, language subscale.

### MASLD and fibrosis

**Fig. 1:**
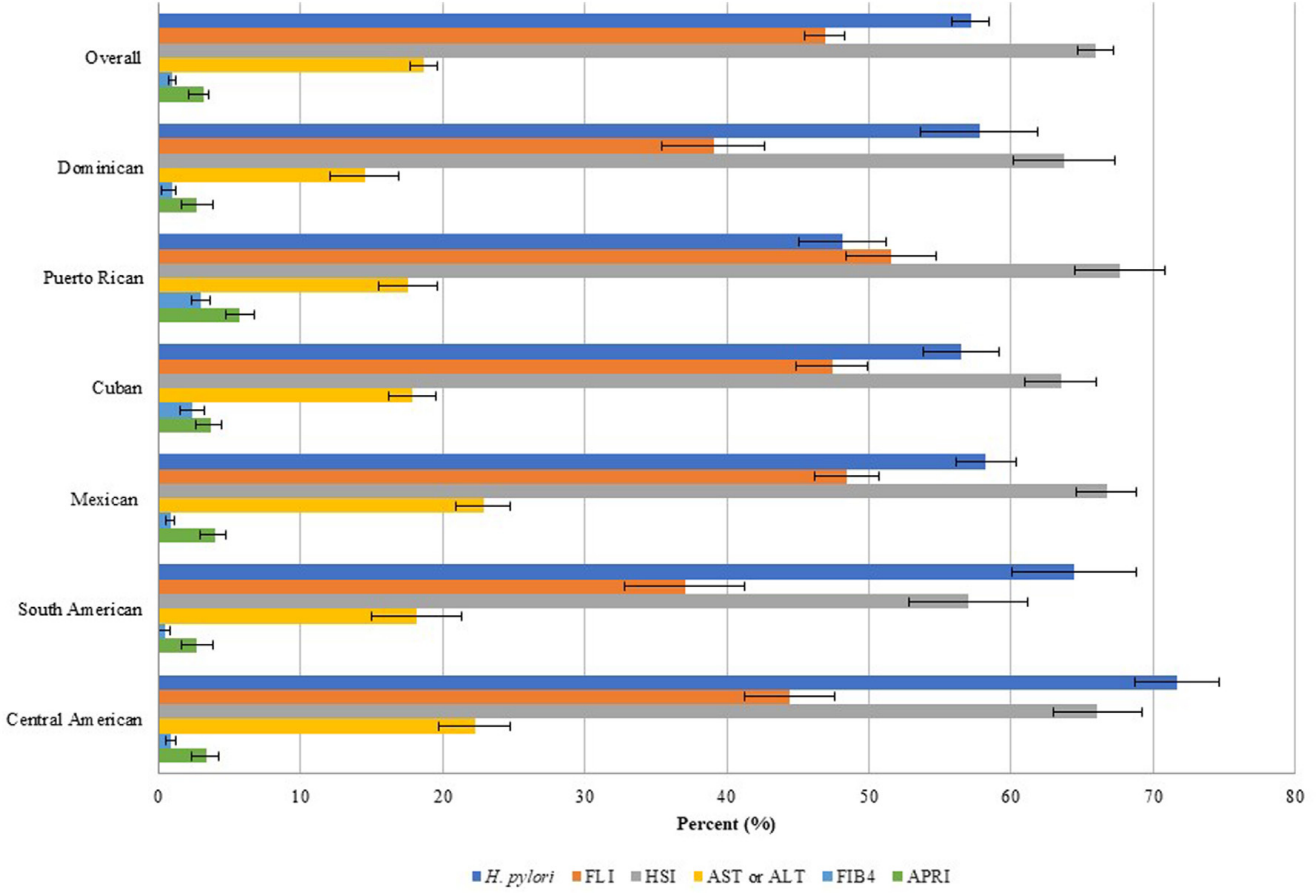
Overall and Hispanic/Latino Heritage-specific weighted prevalences of *Helicobacter pylori* and liver diseases. Footnote: MASLD as determined by elevated FLI and HSI. Suspected MASLD as determined by elevated AST or ALT.

The prevalence of MASLD varied depending on the index used (Fig. 1). Overall, 46.9% of the participants had an elevated FLI (ranging from 37.1% among persons of South American heritage to 51.6% among persons of Puerto Rican heritage), 65.2% had an elevated HSI (57.0% among persons of South American heritage to 67.7% among persons of Puerto Ricans heritage), and 19.8% had an elevated ALT or AST level (14.5% among persons of Dominican heritage to 22.8% among persons of Mexican heritage). In addition, the overall prevalences of liver fibrosis as determined by elevated FIB-4 was 1.5% (ranging from 0.5% among persons of South American heritage to nearly 3.0% among persons of Puerto Rican heritage) and 4.0% had an elevated APRI (2.6% among persons of South American heritage to 5.7% among persons of Puerto Rican heritage) (Fig. 1).

The seroprevalence of *H. pylori* was 57.0%, which differed by Hispanic/Latino heritage (Fig. 1). Notably, persons of Puerto Rican heritage had the lowest seroprevalence of *H. pylori* (48.1%) while persons of Central American heritage had the highest seroprevalence (71.7%).

Crude prevalences for the presence of liver diseases and metabolic conditions by seropositivity of *H. pylori* are presented in Table 3.

**Table 3:** Crude prevalences for the presence of liver disease and metabolic conditions by seropositivity of *Helicobacter (H.) pylori*.

	Seropositive *H. pylori* crude prevalence of liver disease and metabolic conditions (%)	Seronegative *H. pylori* crude prevalence of liver disease and metabolic conditions (%)
**Liver disease**		
Elevated fatty liver index	50.7 (4944/9761)	48.9 (3020/6177)
Elevated hepatic steatosis index	70.2 (6907/9840)	66.5 (4143/6233)
Suspected MASLD	20.2 (1999/9876)	19.6 (1229/6263)
Elevated FIB-4 score	1.7 (170/9786)	2.0 (123/6216)
Elevated APRI	4.3 (417/9786)	4.5 (280/6218)
**Metabolic condition**		
Obesity	42.2 (4153/9844)	42.5 (2652/6241)
Abdominal obesity	60.8 (5990/9851)	60.9 (3799/6240)
Diabetes	20.6 (2038/9877)	18.2 (1138/6267)
Metabolic syndrome	39.4 (3894/9877)	35.5 (2225/6267)

Abbreviation: *H*, *Helicobacter.*

Denominators may vary due to the exclusion of participants with missing information for each condition.

Prevalences are unweighted.

### Metabolic conditions

**Fig. 2:**
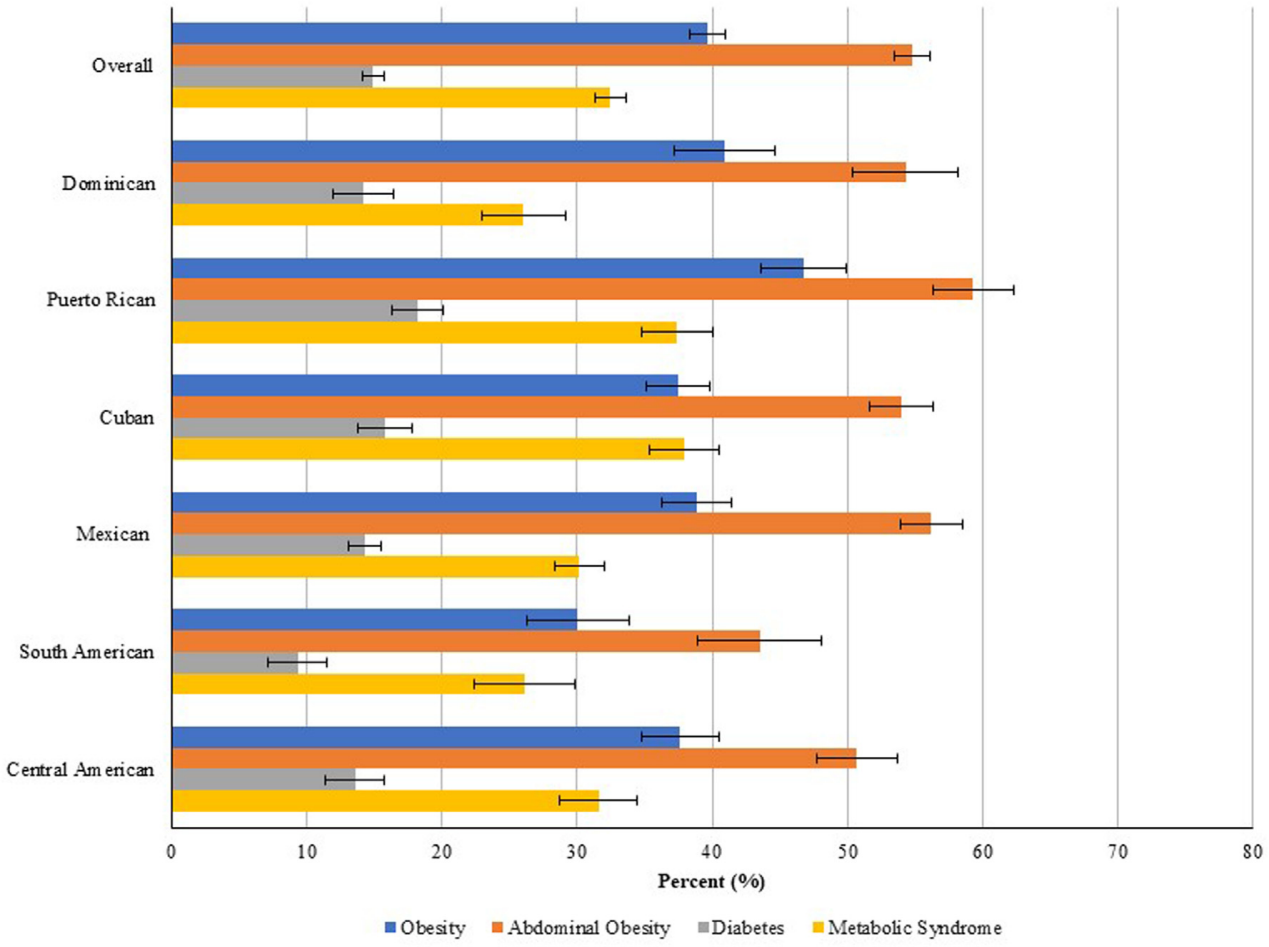
Overall and Hispanic/Latino heritage-specific weighted prevalences of metabolic conditions.

Overall, the prevalences of obesity and abdominal obesity were 39.7% and 54.8%, respectively, ranging from 30.1% (obesity) and 43.5% (abdominal obesity) among persons of South American heritage to 46.7% (obesity) and 59.3 (abdominal obesity) among persons of Puerto Rican heritage (Fig. 2). Furthermore, the prevalence of diabetes was 14.9% overall (9.3% among persons of South American heritage to 18.2% among persons of Puerto Rican heritage), while the overall prevalence of metabolic syndrome was 32.5% (ranging from 26.1% among persons of Dominican heritage to 37% among persons of Cuban and Puerto Rican heritage).

### Associations of liver diseases and *H. pylori* seropositivity

**Fig. 3:**
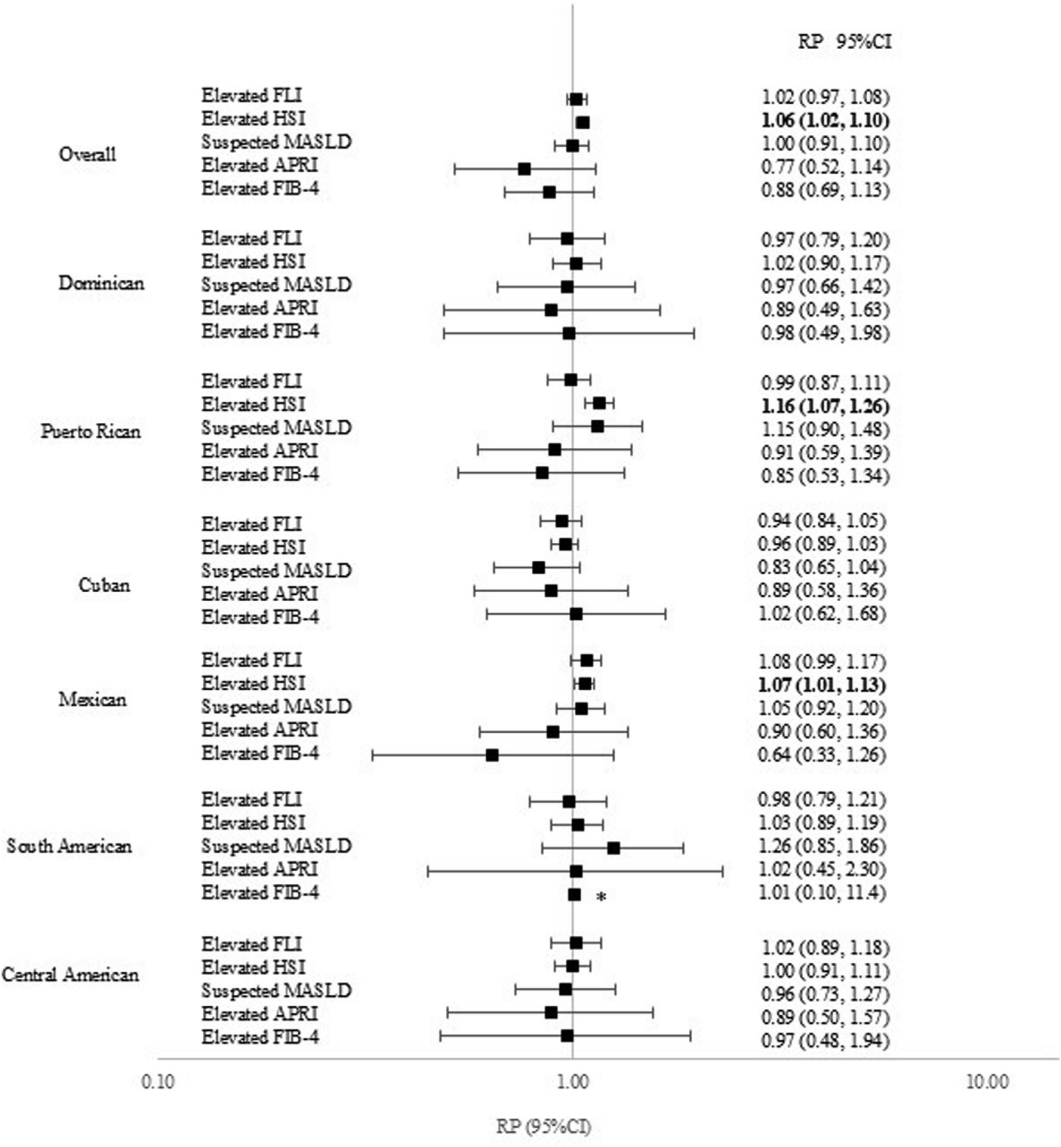
Overall and Hispanic/Latino Heritage-specific Ratios of Adjusted Prevalences (RP) and 95% Confidence Intervals (CI) for *Helicobacter pylori* Seropositivity and Liver Conditions. Footnote: ∗95% CI: 0.10, 11.4 not displayed.

The multivariable-adjusted logistic regression analysis found a modest association between elevated HSI and *H. pylori* seropositivity (RP: 1.06, 95% CI: 1.02, 1.10) (Fig. 3). In addition, adding interaction terms between *H. pylori* seropositivity and the covariates in the final model revealed an interaction close to statistical significance between *H. pylori* seropositivity and Hispanic/Latino background for elevated HSI (p value = 0.06). Analysis by heritage found that this association was observed among persons of Puerto Rican (RP: 1.16, 95% CI: 1.07, 1.26) and Mexican heritages (RP: 1.07, 95% CI: 1.01, 1.13) (Fig. 3). No associations were observed for other liver indices or fibrosis score.

### Association of metabolic conditions and *H. pylori* seropositivity

**Fig. 4:**
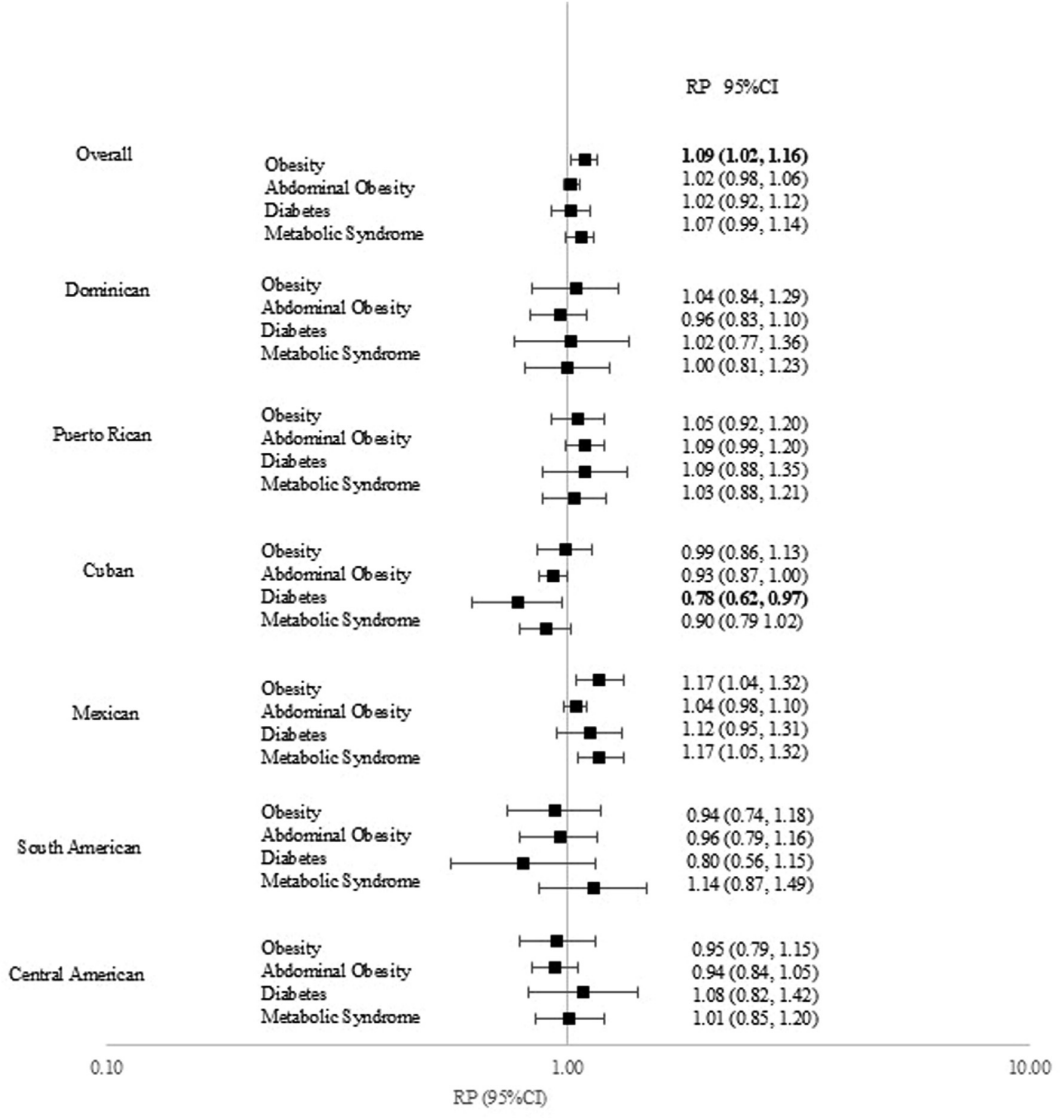
Overall and Hispanic/Latino heritage-specific ratios of adjusted prevalences (RP) and 95% confidence intervals (CI) for *Helicobacter pylori* seropositivity and metabolic conditions.

Overall, a modest association between *H. pylori* seropositivity and obesity was observed (RP: 1.09, 95% CI: 1.02, 1.16) (Fig. 4). Adding interaction terms in the final model revealed an interaction close to statistical significance between *H. pylori* seropositivity and Hispanic/Latino background for diabetes (p value = 0.06). In the stratified analysis by Hispanic/Latino heritage, an inverse association was observed between *H. pylori* seropositivity and diabetes among persons of Cuban ancestry (RP: 0.78, 95% CI: 0.62, 0.97).

## Discussion

The current study confirmed a high burden of liver disorders, metabolic conditions and *H. pylori* seropositivity in the U.S. Hispanic/Latino population. Prevalence of *H. pylori* in HCHS/SOL is higher compared to other populations. In addition, our study found evidence of a modest positive association between MASLD and *H. pylori* seropositivity. Stratification by heritage found that the *H. pylori*-MASLD association was significant among individuals of Puerto Rican or Mexican heritage. Furthermore, a positive association between obesity and *H. pylori* seropositivity was observed, overall, while an inverse association between diabetes and *H. pylori* seropositivity was found among persons of Cuban heritage. No associations were observed for other liver disorders and metabolic conditions.

The results of the current study are consistent with other observational studies, predominantly from Asia, that have reported an association between *H. pylori* and fatty liver disease, as well as other metabolic disorders.^9^^,^^18^^,^^19^ Two recent meta-analyses, reported a positive association between *H. pylori* and NAFLD (pooled ORs 1.26–1.28).^6^^,^^20^ Both meta-analyses reported little to no heterogeneity across studies. Further examination from the meta-analyses showed that the *H. pylori*-NAFLD association was more evident with increased severity of disease.^20^ In contrast, we found no association between *H. pylori* and the liver fibrosis indices as markers of disease severity. In addition, a recent randomised controlled trial investigating the effect of *H. pylori* eradication among persons with NAFLD found significant reductions in metabolic indices and hepatic steatosis transient elastography values 1 year after treatment. The authors hypothesised that *H. pylori* eradication therapy may decrease the severity of NAFLD by reducing inflammatory indicators in the system.^21^ In addition, a meta-analysis that investigated the relationship between *H. pylori* seropositivity and obesity in China, reported a positive association with a pooled OR = 1.20 (95% CI: 1.13–1.28).^19^

In addition to the studies that found a NAFLD-*H. pylori* association, several studies have also reported no association. A cross-sectional study in China (n = 21,456) found no association between *H. pylori* infection and ultrasound-defined NAFLD among healthy individuals.^22^ In Brazil, a cross-sectional study observed that *H. pylori* infection was not significantly associated with NAFLD determined by biopsy among individuals undergoing bariatric surgery.^23^ In Japan, a cross-sectional study (n = 13,737) reported that *H. pylori* infection was not associated with ultrasound-defined NAFLD among either sex.^24^ A study that conducted a Mendelian randomisation analysis of *H. pylori* infection and NAFLD reported no evidence of a relationship.^25^ The study also observed no significant correlation between *H. pylori* and relevant metabolic characteristics of NAFLD including triglyceride levels, low- and high-density lipoprotein cholesterol, fasting blood glucose, and BMI. A previous study in Guatemala also found no associations between *H. pylori* and other species of *Helicobacter* with NAFLD or related conditions. However, seropositivity for *H. pylori* antigens CagA and VacA was associated with NAFLD. Other *H. pylori* antigens showed significant associations with specified metabolic conditions, including obesity.^8^

The precise mechanism underlying our findings is not well established. It has been suggested that *H. pylori* may contribute to a systemic low grade chronic inflammation resulting in an elevation of proinflammatory cytokines and hormonal abnormalities, playing a larger and long-term role in the development of steatotic liver disease and insulin resistance.^26^ In addition, it has been hypothesised that *H. pylori* could influence the development of steatotic liver disease by hormonal effects. For example, *H pylori* has been shown to regulate metabolic hormones (e.g., leptin, ghrelin, etc.) and adipose tissue hormones such as adiponectin that can contribute to the development of steatotic liver disease.^27^ Recent evidence has indicated that gut microbiome might also play an important role in the development of steatotic liver disease. *H. pylori* modulates the composition of the gut microbiota via dysbiosis, which could increase gut permeability and facilitate bacterial endotoxin passage via the portal vein to the liver.^28^ Others have hypothesised the existence of a multiple-hit hypothesis in which *H. pylori* and the gut microbiome play an interrelated role in the development of steatotic liver disease, which involves multiple insults acting together in genetically predisposed individuals.^29^

Previous studies have reported that *H. pylori* infection is associated with diabetes, however the relationship remains controversial.^30^ It has been suggested that *H. pylori* causes insulin resistance and chronic inflammation that could contribute to the disease.^31^ It is unclear why our study found an inverse association between *H. pylori* and diabetes among Cuban heritage. Thus, further research is needed to confirm these results.

### Strengths and limitations

The current study has several strengths, including a large, diverse, and well-characterized sample of Hispanics/Latinos living in the U.S. Although HCHS/SOL was not designed to be nationally representative, currently it is the most generalizable cohort of Hispanics/Latinos in the U.S. In addition, our analysis accounted for a wide variety of sociodemographic, clinical and lifestyle factors. An important limitation of the current study is its cross-sectional design which precludes the ability to examine a temporal relationship between *H. pylori* with MASLD and metabolic conditions. Future longitudinal studies are warranted to explore the temporal sequence and potential causal pathways underlying these associations. Another limitation is that liver steatotic indices, are not as specific or as sensitive as other diagnostic tools such as magnetic resonance imaging, ultrasound or transient elastography (TE). The performance of the indices, however, has been validated previously in other populations. Moreover, recently good agreement between MASLD by liver indices (FLI and HSI) and MASLD by TE was found in the U.S. NHANES. The percent agreement for FLI and TE was 75.1% and for HSI and TE was 74.3%.^2^ Furthermore, while the *H. pylori* assay has not been specifically validated within our study population, it has undergone clinical validation demonstrating high sensitivity and specificity, and a robust area under the curve of 0.978, indicating excellent diagnostic accuracy.^31^ The current study did not examine *H. pylori* strains or other species of *Helicobacter* that were found to be associated with steatotic liver and metabolic conditions in a previous study.^8^ Finally, we were unable to analyse CagA or VacA serology, which can provide insights into *H. pylori* virulence, this could be a valuable addition to future studies investigating the mechanisms underlying associations between *H. pylori* and MASLD.

### Conclusions

In conclusion, our large and diverse study of the U.S. Hispanic/Latino population found evidence of a cross-sectional association of *H. pylori* with MASLD, overall and among some Hispanic/Latino heritage groups. An association between *H. pylori* and obesity was also observed, overall. Underlying mechanisms responsible for the association are not fully elucidated, thus further studies are warranted to confirm the findings and explain potential mechanisms.

## Contributors

Conceptualization: CSA, KAM. Data acquisition: RCK, MCC, MLA-S, MD, OG-B, CRI, MSP, BT, GAT. Data verification and curation: CSA. Data analysis/Interpretation: CSA, BIG, KAM. Writing original draft: CSA. Reviewing and editing: All authors. All authors approved the final manuscript. CSA and KAM had full access to all data in the study. CSA and KAM had final responsibility for the decision to submit for publication.

## Data sharing statement

The HCHS/SOL fully supports data sharing with outside HCHS/SOL investigators through processes internal to the study, based on a Data and Materials Distribution Agreement (DMDA) to protect the confidentiality and privacy of the HCHS/SOL participants and their families. HCHS/SOL internal processes for new investigators to get involved with the study are described at %%https://sites.cscc.unc.edu/hchs/New%20Investigator%20Opportunities, accessed on 4 October 2021. Alternatively, de-identified data from HCHS/SOL and the SOLNAS ancillary study are publicly available at BioLINCC for the subset of the study cohort that authorised general use of their data at the time of informed consent (https://biolincc.nhlbi.nih.gov/studies/hchssol/, accessed on 4 October 2021).

## Author's disclaimer

The authors alone are responsible of the views expressed in this publication. Their opinions do not represent the official position of the National Cancer Institute, National Institute on Minority Health and Health Disparities, or the U.S. federal agencies.

## Declaration of interests

Dr. Daviglus reports research grants from the National Institutes of Health (NIH) during the conduct of the study. Dr. Kaplan was supported by 5R01HL144707 from the NIH/HNLBI and 1R01DK134672 from the NIH/NIDDK during the conduct of the study. Dr. Thyagarajan reports grants from the NIH/NHLBI during the conduct of the study. Dr. Talavera reports grants from the NIH/NHLBI during the conduct of the study. Dr. Avilés-Santa was the NIH/NHLBI Project Officer for the parent study. NHLBI is the lead NIH funder of the HCHS/SOL. She currently works at NIMHD, which is a co-founder of the HCHS/SOL. The other authors have no competing interests.
